# Human behavior determinants of exposure to *Anopheles* vectors of malaria in Sumba, Indonesia

**DOI:** 10.1371/journal.pone.0276783

**Published:** 2022-11-14

**Authors:** Ismail E. Rozi, Lepa Syahrani, Dendi H. Permana, Puji B. S. Asih, Anggi P. N. Hidayati, Sully Kosasih, Farahana K. Dewayanti, Rifqi Risandi, Siti Zubaidah, Michael J. Bangs, Claus Bøgh, John P. Grieco, Juan E. Baus, Evercita Eugenio, April Monroe, Fang Liu, Nicole L. Achee, Din Syafruddin, Neil F. Lobo

**Affiliations:** 1 Eijkman Research Center for Molecular Biology, National Research and Innovation Agency, Jakarta, Indonesia; 2 Public Health and Malaria Control, PT Freeport Indonesia, International SOS, Kuala Kencana, Papua, Indonesia; 3 Faculty of Agriculture, Department of Entomology, Kasetsart University, Bangkok, Thailand; 4 The Sumba Foundation, Public Health and Malaria Control, Bali, Indonesia; 5 Department of Biological Sciences, Eck Institute for Global Health, University of Notre Dame, Notre Dame, Indiana, United States of America; 6 Sandia National Laboratories, Albuquerque, New Mexico, United States of America; 7 Johns Hopkins Center for Communication Programs, Baltimore, Maryland, United States of America; 8 Applied and Computational Mathematics and Statistics, University of Notre Dame, Notre Dame, Indiana, United States of America; 9 Department of Parasitology, Faculty of Medicine, Hasanuddin University, Makassar, Indonesia; Fundação Oswaldo Cruz Centro de Pesquisas René Rachou: Fundacao Oswaldo Cruz Instituto Rene Rachou, BRAZIL

## Abstract

Malaria vector control interventions in Sumba, Indonesia, have not been able to eliminate malaria. Human drivers of exposure to *Anopheles* bites were investigated as part of a larger clinical trial evaluating the impact of a spatial repellent product on malaria incidence. Human behavioral observations (HBOs) evaluating temporal and spatial presence, sleeping behaviors, and insecticide treated net (ITN) use, were collected parallel to entomological collections—indoor and outdoor human landing catches (HLCs), and house hold surveys. Data demonstrates that mosquito access to humans, enabled by structurally open houses, is evident by the similar entomological landing rates both inside and outside households. The presence of animals inside houses was associated with increased mosquito entry–however, the number of humans present inside houses was not related to increased mosquito landing. Analyzing mosquito landing rates with human behavior data enables the spatial and temporal estimation of exposure to *Anopheles* bites, accounting for intervention (ITN) presence and usage. Human behavior adjusted exposure to *Anopheles* bites was found to be highest in the early in the evening, but continued at lower levels throughout the night. Over the night, most exposure (53%) occurred when people were indoors and not under the protection of nets (asleep or awake) followed by exposure outside (44%). Characterized gaps in protection are outdoor exposure as well as exposure indoors–when awake, and when asleep and not using ITNs. Interestingly, in the primary trial, even though there was not a significant impact of the spatial repellent on vector biting rates by themselves (16%), when factoring in human behavior, there was approximately 28% less exposure in the intervention arm than in the placebo arm. The treated arm had less human behavior adjusted bites in all spaces evaluated though there was proportionally higher exposure indoors. This analysis points to the importance of using HBOs both towards understanding gaps in protection as well as how interventions are evaluated. To mitigate ongoing transmission, understanding context specific spatial and temporal exposure based on the interactions of vectors, humans and interventions would be vital for a directed evidence-based control or elimination strategy.

## Introduction

The Indonesian national strategic plan to control malaria is conducted through the malaria elimination program. Intervention activities include early diagnosis with prompt and accurate treatment, surveillance, and vector control [1,2]. The primary vector control interventions include the distribution of insecticide-treated bed nets (ITNs), indoor residual spraying (IRS), and larval source management (LSM) that includes larvicide, biological control, and environmental management. However, the decentralized system with various local (district) priorities in health financing and implementation may pose a barrier to the elimination agenda especially in the eastern part of Indonesia—since malaria may not be either considered or be a priority [2–4]. Environmental and human drivers both expose or protect people from malaria. Environmental drivers of malaria that determine vector population include the ecology, weather, and temperature, while human drivers include local cultural practices and behavior [5,6], implemented interventions and their usage, house construction, and mobility. For example, even though malaria was eliminated in Sabang Municipality, Aceh, Indonesia, a comprehensive analysis including human, vector and other components of the transmission system demonstrated a significant potential for reintroduction of malaria. A mobile and susceptible population resulted in high vulnerability, the presence of endemic populations of malaria vectors including *Anopheles sundaicus*, *An*. *minimus*, *An*. *aconitus* and *An*. *dirus* [7] resulted in high receptivity, while local factors that specifically contributed to malaria in this area include the importation of malaria infections, as well as *Plasmodium knowlesi* transmission stemming from long-tailed macaques and endemic *Anopheles* in the Leucosphyrus group [8].

Although more than half of districts have been declared malaria free, Indonesia is still one of nine malaria-endemic countries in the South-East Asia region, and accounts for 21% of the region’s reported cases and 16% of malaria deaths [1,9]. In order to achieve the goal of malaria elimination in Indonesia by 2030, a strategy that focuses on local drivers of transmission may be required. A successful implementation of this approach was demonstrated in the Purworejo area of Central Java, Indonesia, where multiple local factors that enable transmission were identified, including drivers of vector populations, locally relevant interventions, the implementation of epidemiological control measures, and the political system [10].

Even though the need for characterizing where and when human and vectors overlap was recognized in the 1960s [11], the incorporation of human behavior has not been included in many studies [12–17]. Key measures include understanding when and where the vector and human overlap occurs as well as understanding the human activities that put people at risk [13]. Activities that may increase risk include routine household and community activities, large scale socio-cultural events, as well as regular livelihood or economy-based activities [18,19].

Most residents in rural Southwest Sumba and West Sumba districts are subsistence agriculturalists such as farming and raising livestock, where night-time activity such as cooking and/or protecting stable animals (pigs and horses), hunting, and fishing is a necessity [20]. Moreover, traditional Sumba houses with open construction allow for vector entry and indoor exposure. The use of ITNs as well as factors that may increase use are not well documented. Documentation of human behaviors in conjunction with vector behaviors would allow insights into local gaps in protection that could lead to continued transmission and limit the impact of intervention efforts such as suboptimal ITN usage [13]. Towards addressing this knowledge gap, data was collected on indoor and outdoor vector biting behavior and corresponding human behaviors including time spent indoors versus outdoors, awake versus asleep, and under the protection of an ITN throughout the night.

## Methods

### Study site

**Fig 1:**
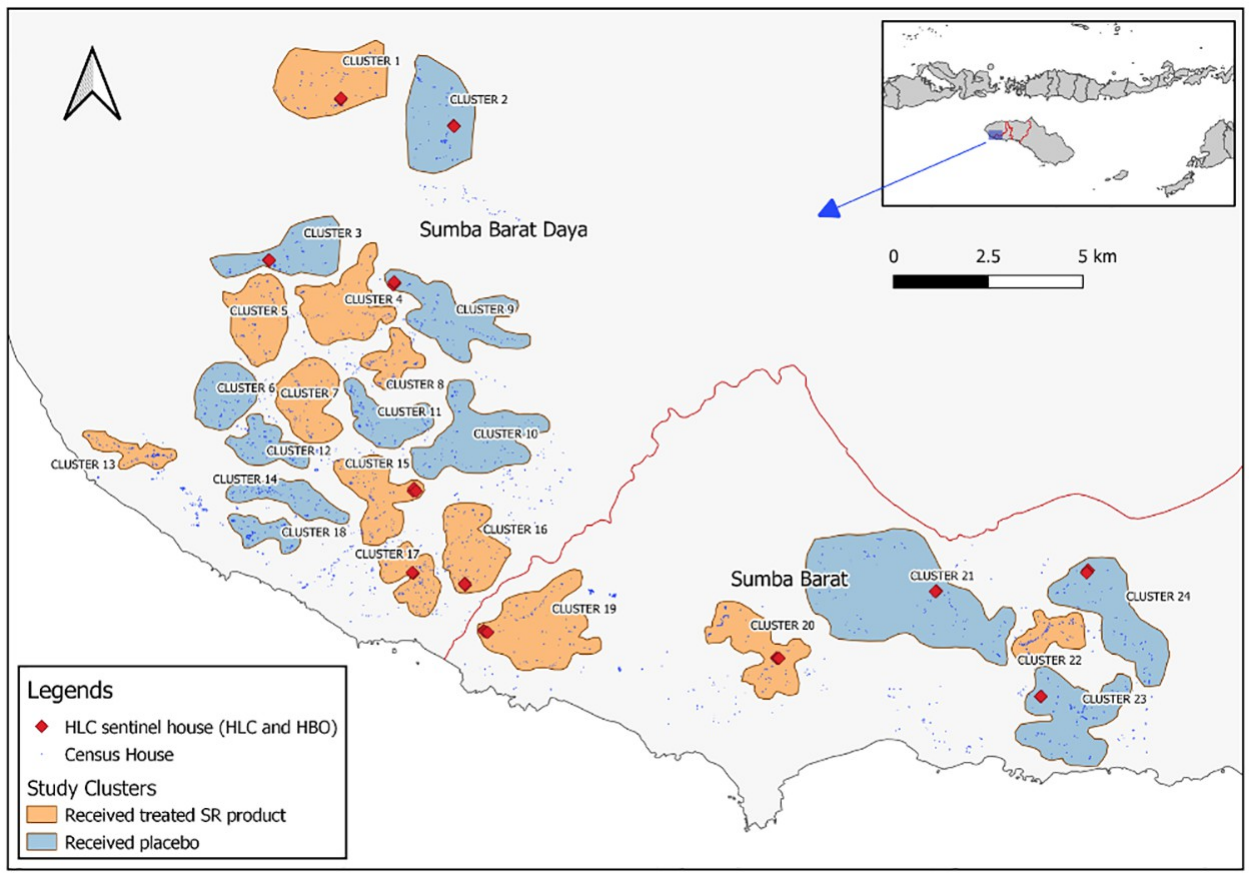
Primary study area. Primary study area with treated clusters in orange and untreated clusters in blue. Diamonds indicate HLC/HBO sentinel houses. The inset indicates the location of the study site in Sumba Island, Nusa Tenggara Timur Province. Map source: Natural Earth (https://www.naturalearthdata.com/).

This study was conducted in parallel with an epidemiological trial to determine the effect of spatial repellents on malaria transmission in Southwest and West Sumba, East Nusa Tenggara, Indonesia [21]. Sumba island, a part of East Nusa Tenggara province, has an area of around 11 thousand square kilometers, an estimated population of more than 755,000 (2015), and is divided into 4 districts. The geography is generally of low elevation and consists of limestone hills. The dry season extends from May to November, and the wet season from December to April. The districts of Southwest Sumba and West Sumba are included in the 22 districts that still had the highest annual parasite incidence (API) in Indonesia in 2019. The dominant malaria parasites in Sumba are *P*. *falciparum* and *P*. *vivax*, with the occasional case of *P*. *malariae*. Studies in Southwest Sumba, West Sumba and Central Sumba districts in 2007 demonstrated malaria seasonal prevalence of 6.83% in the wet season and 4.95% in the dry season [22]. In the wet season *P*. *falciparum* accounted for 70% of infections while in dry season *P*. *falciparum* and *P*. *vivax* were present in equal proportion. Malaria vectors detected from previous studies include *An*. *aconitus*, *An*. *annularis*, *An*. *barbirostris*, *An*. *flavirostris*, *An*. *maculatus*, *An*. *sundaicus*, *An*. *tesellatus*. *An*. *subpictus*, and *An*. *vagus* [21–25]. There were more than 430,000 residents in the two study districts occupying 92 villages and 13 small-sized towns [26,27]. Thirteen village groups, with populations ranging from 1,067 to 3,904 (avg. 2,132), served as the 12 study clusters included in this study (Fig 1).

### Study population

This study included human participants that are residents of West and Southwest Sumba Districts, East Nusa Tenggara Province, Indonesia (Fig 1). Most participants are of Sumba Ethic groups that adopt both traditional beliefs and Christianity. The majority of the study participants are subsistence farmers, who also perform animal husbandry and fishing. Livestock includes pigs, buffalos that are traditionally and culturally used as currency for social and cultural activities [21,28].

### Human Landing Catch (HLC) collections

HLCs were conducted in 12 clusters continuously every two weeks from June 2015 to April 2018 with six clusters each from placebo (untreated) and intervention (treated) clusters [21]. For the adjusted biting rate analysis, only HLC data that overlapped with human behavioral observations (HBOs) were used (February 2018 to April 2018), and included five HLC periods from the parent study intervention period (HLC #I48 to #I52). Four sentinel houses within each of the 12 clusters were selected for paired (indoor and outdoor/house verandah) HLC collections. Mosquito collections were conducted from 1800 h until 0600 h the next day. At the end of each HLC, a household questionnaire was conducted that documented the presence of fire and/or smoke, and animals both inside and outside the structure during the HLC (S1 File. HLC Questionnaire). Human landing rates (HLR) were used as a proxy for human biting rates (HBRs) and were calculated as bites per person per night (bpn) or bites per person per night (bpn) for the location of collection (inside and outside).

### Human Behavioral Observations (HBOs)

HBOs were conducted hourly from 1800 h to 0600 h alongside entomology collections in HLC households. HBO data was collected during five HLC periods (February 2018 to April 2018) in 12 clusters—resulting in data from 48 houses over five nights each (S2 File. HBO Questionnaire). HBOs focused on temporal (hourly, over the night), location (domestic or peri-domestic) and bed net usage. HBO data was limited to the perimeter of the sentinel structure (usually less than 20 m around the structure). Data was collected by the HLC volunteer on paper questionnaires at the end of each HLC hour, with spot-checks conducted by a supervisor, and double data entry checks after data collection towards ensuring quality. The HLC volunteers were part of the community and their presence was excluded from the HBO data and analysis.

### Household and bed net (ITN) surveys

Household and ITN surveys were conducted in every household during household enrollment in the parent study [21]. The household questionnaire collected data on structure materials, practices against mosquito bites, the presence of livestock and ITN presence (S3 File. Household and ITN questionnaire).

### Analysis

Analysis of quantitative data was carried out using Microsoft Office Excel basic functions and open-source software, RStudio version 1.3.1056 based on R version 4.0.2 [29,30]. Clustering of house type analysis was done using the klaR package clustering of categorical variables [31]. The effect of fire/smoke (indoor or outdoor) and large animals (indoor or outdoor) on mosquito landing rates, were separated into time periods (1800h – 0000h, and 0000h to 0600h), and were evaluated using Kruskal-Wallis test. With mosquito landing rates being normally distributed, the number of people in each house were evaluated using a regression analysis towards determining if they had an effect on mosquito numbers. Human behavior-adjusted biting rates and the protections afforded by ITNs were generated using HLRs, HBOs and ITN survey data as in Monroe et. al, 2020 [12,13].

### Ethical review

Ethical review and approval for this study was granted by the Ethics Committee (EC) of the Faculty of Medicine, Universitas Hasanuddin, Indonesia (Protocol #UH14070385), the University of Notre Dame, USA (Protocol #14-01-1448), and endorsed by the Eijkman Institute Research Ethics Committee, Jakarta, Indonesia.

## Results

### Demographic characteristics

All households (n = 2910) were characterized before inclusion in the parent study [21] (Table 1). Most household structures enrolled were made from traditional materials including bamboo and wood with thatch roofs. A clustering analysis of wall, roof and floor materials (household dataset n = 2842) demonstrated an optimal characterization into four house type groups—Type 1: The traditional house type (n = 1985, 69.85%) with bamboo structure and a bamboo floors; Type 2: (n = 339, 11.93%) bamboo structure with dirt floors and thatch roofs (some were replaced with metal); Type 3 (n = 297, 10.45%) were households with concrete floors and metal roof, with walls made of are plaster or bamboo; and Type 4 (n = 221, 7.78%) consisted of wood houses with thatch or metal roofs. The traditional Sumba house has floors made of bamboo (Type 1), with a raised floor (mean floor height is 145.3 cm ± 38.8 cm) under which livestock is reared. Type 1 houses do not have doors or windows, and have open exits—one each at the front and rear of the house. These structures have hollow bamboo walls. The other types of houses have doors and windows, without screening material (only four houses had windows with screening material). Overall, 2864 households (98.5%) had open eaves. Household cooking is usually conducted at an indoor central location using firewood (84.3% of households), resulting in the presence of smoke indoors. Food preparation for livestock was performed outdoors in 34.9% of households. Approximately 81.3% of the households did not have a source of electricity. All structures are open and permeable to mosquito entry with clear demarcations of inside versus outside spaces irrespective of house type.

**Table 1:** Household survey results (total household n = 2910).

Variable	Category	Total (percentage)
**Wall type**	Bamboo	2399 (82.5%)
	Plaster	229 (7.9%)
	Wood	211 (7.3%)
	Split wood	31 (1.1%)
	Others (concrete, brick, etc)	39 (1.2%)
**Roof type**	Thatch	2273 (78.1%)
	Metal	632 (21.7%)
	Tiles and sticks	4 (0.1%)
**Floor type**	Bamboo	2155 (74.1%)
	Concrete	365 (12.5%)
	Dirt	318 (10.9%)
	Tiles	41 (1.4%)
	Wood	21 (0.7%)
	Other	9 (0.3%)
**Eaves opened**	Yes	2864 (98.5%)
	No	45 (1.5%)
**Door number**	Mean (SD)	2.0 (± 0.4)
**Window number**	Mean (SD)	0.6 (± 1.5)
**Screened windows**	Yes	4 (0.1%)
	No	2706 (99.4%)
**Floor height for Sumba traditional bamboo house (in cm) (subset n = 1985)**	Mean (SD)	145.3 (± 38.8)
**Usually fire/burning indoor**	Yes	2295 (84.3%)
	No	427 (15.7%)
**Usually fire/burning outdoor**	Yes	950 (34.9%)
	No	1772 (65.1%)
**Electrical source**	Yes	507 (18.6%)
	No	2214 (81.3%)
**IRS**	Never	2500 (98.1%)
	Don’t know	45 (1.8%)
	< 3 months	1 (0.0%)
	3–6 months	0 (0.0%)
	> 6 months	3 (0.1%)
**Protection from mosquito bite**	Coil (included emanator)	2 (0.1%)
	Other	3 (0.1%)
	Not used	2717 (99.8%)
**Livestock indoor/under the house**	Has animal	2040 (74.9%)
	Large animals	1952 (71.7%)
	Fowl	1437 (52.8%)
	Others	14 (0.5%)
	No animal	678 (24.9%)
**Livestock outdoor**	Has animals	848 (31.1%)
	Large animals	764 (28.1%)
	Fowl	530 (19.5%)
	Others	4 (0.1%)
	No animal	1880 (69.0%)

### Drivers of human landing rates (HLR)

**Fig 2:**
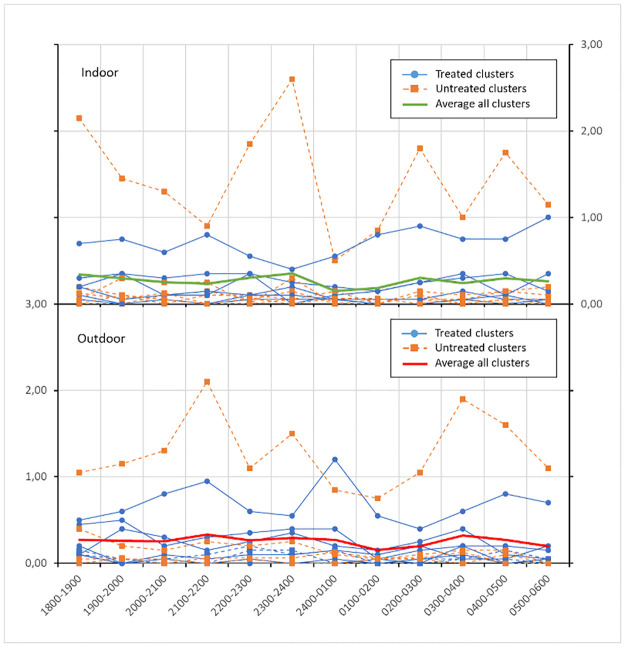
Indoor (A) and outdoor (B) HLRs by cluster over the course of the night. Though there were mosquitoes present throughout the night, there was high heterogeneity in mosquito landing rates between clusters. The solid green and red line indicate average HLRs indoors and outdoors.

A total of 1488 female *Anopheles* mosquitoes were caught during these HLCs, of which 833 came from untreated clusters and 655 from treated clusters. There was significant variation between clusters with cluster 2 sampling the most (n = 655) and cluster 15 the least (n = 15) (Table 2). Equal proportions of *Anopheles* were seen landing indoors and outdoors with no significant difference in total mosquitos caught between indoor and outdoor collections in either arm (Welch Two Sample t-test result p-value = 0.8993). *Anopheles* were host-seeking on humans throughout the night, both indoors and outdoors, in all clusters, although there was cluster-specific variation seen (Fig 2).

**Table 2:** Total collected *Anopheles* mosquitoes during HLCs per cluster (treated cluster: Shaded; untreated cluster: Unshaded).

Cluster	HLC #I48	HLC #I49	HLC #I50	HLC #I51	HLC #I52	Total
01	1	4	4	9	3	21
02	78	66	242	171	98	655
03	2	5	8	17	9	41
09	5	3	13	2	1	24
15	0	1	0	0	14	15
16	75	23	94	74	70	336
17	20	11	7	4	6	48
19	7	28	34	26	16	111
20	26	35	13	42	8	124
21	0	1	No HLC ^a^	8	10	19
23	13	13	5	30	14	75
24	5	0	6	8	0	19
Total Treated	129	102	152	155	117	655
Total Untreated	103	88	274	236	132	833
ALL	232	190	426	391	249	1488

^a^ No HLC activities.

The number of *Anopheles* per cluster per night varied significantly (min = 0, max = 242, mean 24.8 ± 42.50) (Table 2). When evaluating mosquito numbers per cluster per total observation (a five-night collection), there was a minimum of 15 and a maximum of 655 mosquitos sampled, with a mean mosquito number of 124 ± 189.90. Each cluster had different landing rates and consequently differential exposure to *Anopheles* bites. The Shapiro-Wilk’s W test on HLRs over the night or just indoor HLRs demonstrates that the data is not normally distributed (p-value of HLR indoor per night is < 2.2e-16). This also indicated that there was no correlation between the number of mosquitoes collected per house and the total number of humans indoors. There was also no correlation between mosquitoes caught and the presence of fire/smoke indoors, fire/smoke outdoors, and animals outdoors (p-values are 0.4971, 0.5519 and 0.3742 respectively). However, there was a positive relationship between the presence of animals indoors and the numbers of mosquitoes caught indoors (p-value = 0.04882) (data not shown). The two data points describing “no animal indoors all night” and “presence of animals indoors all night” demonstrated that houses with animals tended to be visited by more mosquitoes.

Malaria vectors in South West Sumba and West Sumba Districts [21,23–25] did not demonstrate species-specific indoor or outdoor human landing preferences (Table 3). Indoor and outdoor biting rates of *An*. *aconitus*, *An*. *annularis*, *An*. *barbirostris*, *An*. *flavirostris*, *An*. *maculatus*, *An*. *tessellatus* and *An*. *vagus* (also including *An*. *sundaicus* and *An*. *subpictus* that consisted of only one mosquito each), were sampled at equivalent rates both indoors and outdoors (Table 3).

**Table 3:** Total number of collected female *Anopheles* mosquito species.

Species	Indoor	Outdoor	Total
*An*. *aconitus*	237	208	445
*An*. *annularis*	42	46	88
*An*. *barbirostris*	40	41	81
*An*. *flavirostris*	94	101	195
*An*. *kochi*	54	60	114
*An*. *leucosphyrus*	2	3	5
*An*. *maculatus*	15	22	37
*An*. *sundaicus*	1	0	1
*An*. *subpictus*	1	0	1
*An*. *tesellatus*	63	74	137
*An*. *vagus*	209	170	379
Total	759	729	1488

### Human behavior

**Fig 3:**
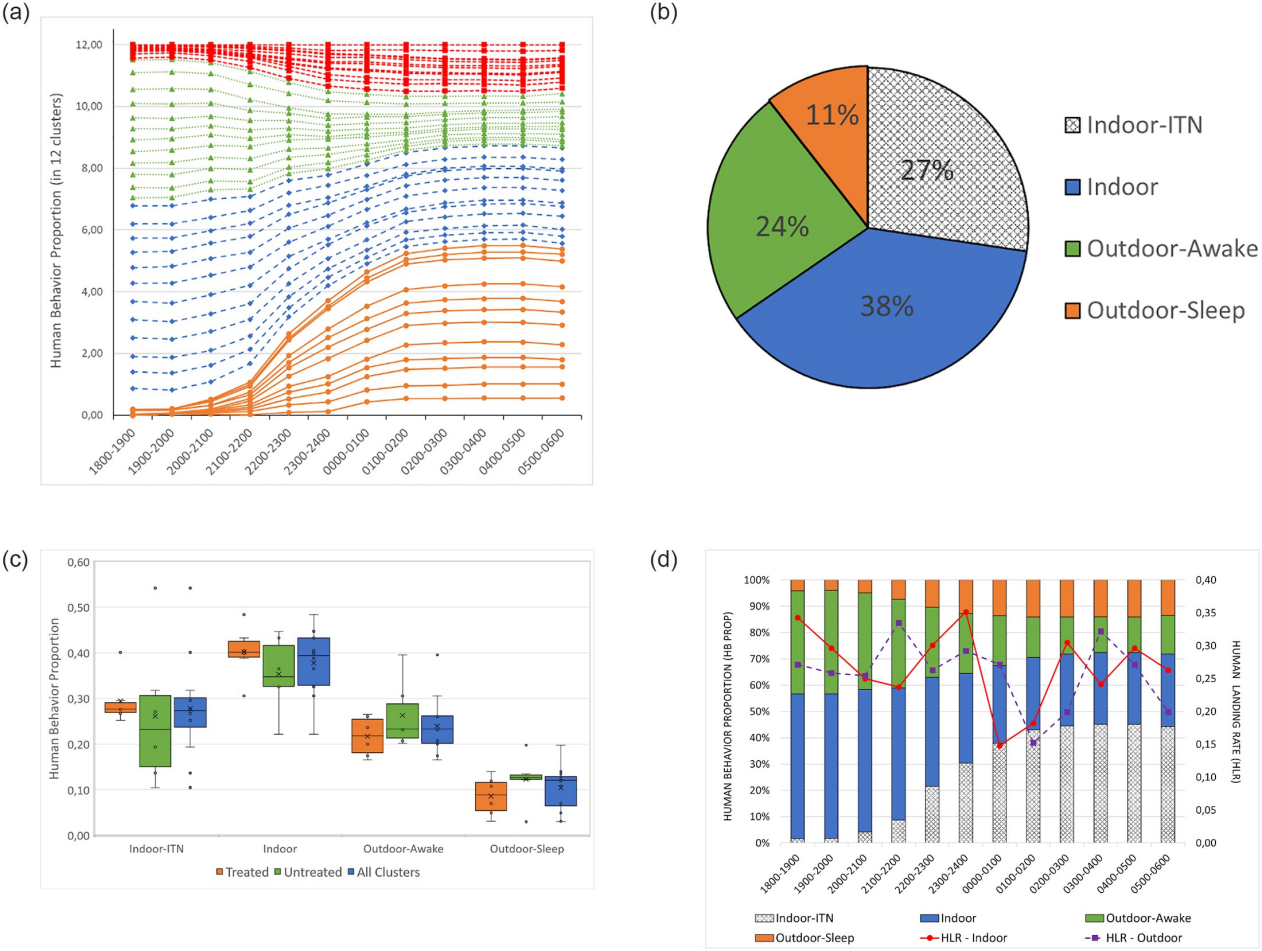
**a. Human behavior proportions from all clusters**. ●: Indoor-ITN, ♦: Indoor, ▲: Outdoor-awake and ■: Outdoor-sleep. **b. Average human behaviors**. Average human behaviors spent in different categories over the course of a night. **c. Average human behavior per treated and untreated clusters. d. Stacked bar graph of the proportions of human behavior overlaid with entomological indoor and outdoor landing rates**.

The communities, in general, move indoors after nightfall (dark) at 1800h and start to go to sleep at about 2000 h (Fig 3a and 3d). Both indoor and outdoor activity during the night is limited since only a proportion (18.6%) of households in the study site have a source of electricity (Table 1). Primary evening and night-time activities before sleep included cooking and eating, where 93.9% of HLC sentinel houses reported the presence of fire/smoke (cooking) indoors after 1800 h (Table 4). Though people spent time outdoors in the peri-domestic area in the evening until midnight, some were documented outdoors throughout the night. ITNs were not utilized outdoors even though about 11% of the population preferred to sleep outdoors on the house verandah. Besides ITNs, only 0.1% of the population used other mosquito bite prevention tools (e.g. mosquito coils) (Table 1).

**Table 4:** HLC household characterization.

**Any fire burning/smoke inside the structure during 1800–2400 h**		
	Yes	93.9%
	No	6.1%
**Any fire burning/smoke inside the structure during 2400–0600 h**		
	Yes	39.1%
	No	60.9%
**Any fire burning/smoke outside the structure during 1800–2400 h**		
	Yes	9.6%
	No	90.4%
**Any fire burning/smoke outside the structure during 2400–0600 h**		
	Yes	7.8%
	No	92.2%
**Any large animals inside (under) the structure during 1800–2400 h**		
	Yes	79.6%
	No	20.4%
**Any large animals inside (under) the structure during 2400–0600 h**		
	Yes	79.6%
	No	20.4%
**Any large animals outside the structure during 1800–2400 h**		
	Yes	39.6%
	No	60.4%
**Any large animals outside the structure during 2400–0600 h**		
	Yes	40.0%
	No	60.0%
**Number of people slept indoor during HLC**		
	Mean	5.6
	SD	1.9
**Number of people slept under ITN during HLC**		
	Mean	2.8
	SD	1.5
**Any efforts to avoid mosquito bites during HLC**		
	ITN	93.0%
	No protection	7.0%

Indoor ITN usage varied from house to house and cluster to cluster (See indoor-ITN in Fig 3a and 3d). Overall, when looking at person time spent in each behavioral category, 24% of time was spent outdoors awake, 11% time was spent outdoors asleep (not under ITN protection), 38% of time was spent indoors not under ITN protection, 27% of time was spent under ITNs (Fig 3b). There remained a proportion of people in all behavioral categories over the course of HBO observations. Fig 3c depicts the box and whisker graphs of the proportion of human behaviors by treated clusters, untreated clusters and all clusters together. The household survey demonstrated large variations in household ITN coverage and use, for example, although the mean number of ITNs per house in cluster #21 was only 2.19, the highest proportion of ITN use was 54%. Paradoxically, the cluster with the highest mean ITN presence per house was 4.28 ITNs in cluster #24, but only 14% actually used the ITNs. The average number of ITNs per household was 1.73 (± 1.27). Fig 3d illustrates the proportion of human behaviors overlaid with the overall vector human landing rate. Although 57% of the population were indoors (including the proportion indoors and using an ITN) by 1800 h, and reached 72% at 0600 h, both indoor and outdoor landing rates remained relatively consistent throughout the night. Excluding the 27% of people indoors using an ITN, the remaining 73% of people remain unprotected from mosquito bites.

### Human behavior adjusted exposure to *Anopheles*

**Fig 4:**
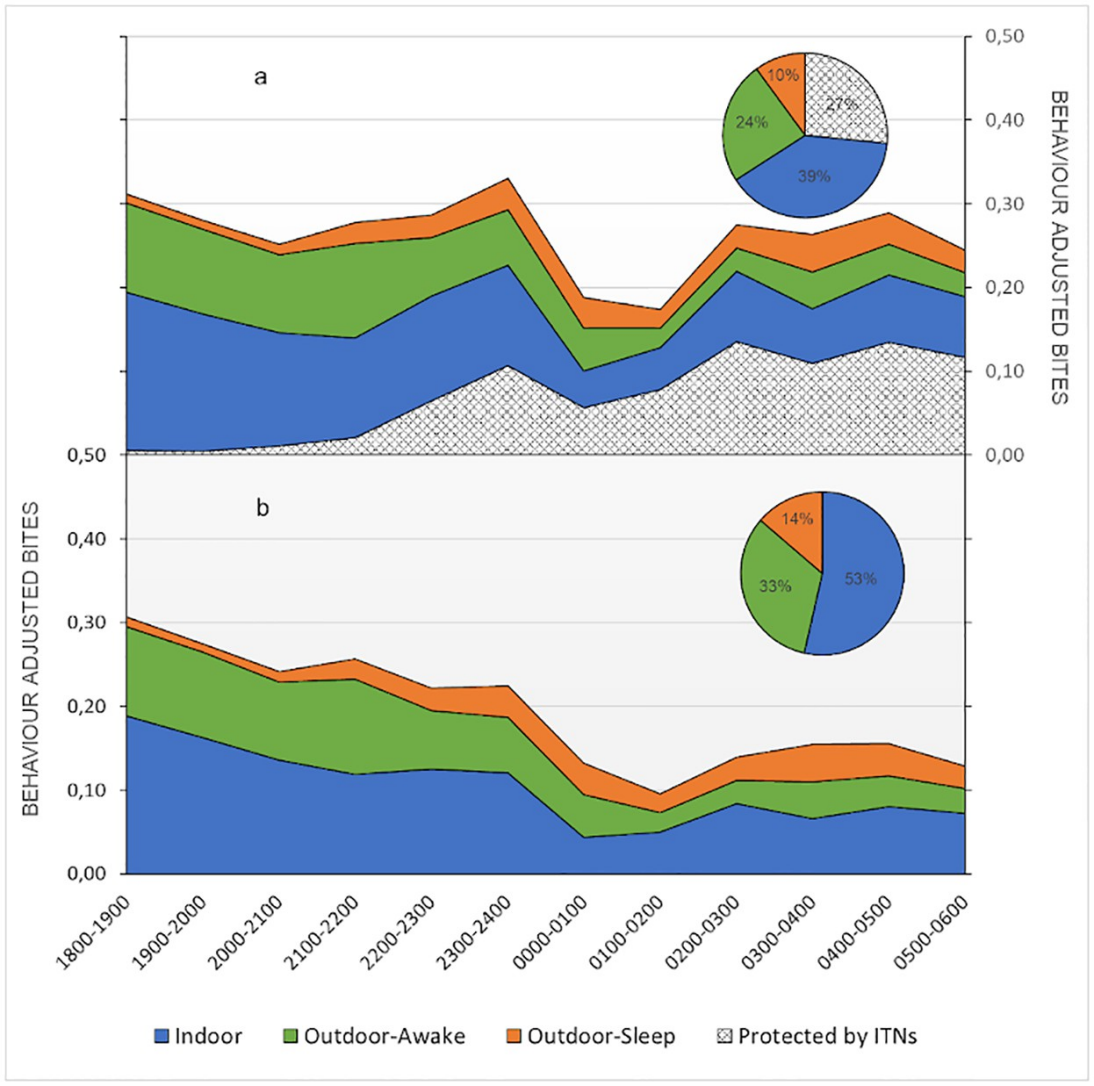
Human behavior adjusted. 4a. Human behavior adjusted temporal exposure and ITN protection over the night with proportional spatial exposure and ITN protection in the inset. ITNs reduce exposure by approximately 27%. 4b. Quantified temporal and spatial (inset) gaps in protection i.e. human behavior adjusted exposure alone, demonstrate that the majority of exposure (53%) occurs when people are indoor and not protected by ITNs.

Towards understanding the relationship between human behavior and vector landing rates, adjusted exposure rates were derived [12]. Overall, as the biting rate drops to a minimum at 2400–0200 h (Fig 2), behavior adjusted bites also decrease (Fig 4a and 4b). The proportion of the population exposed to mosquito bites was highest at early in the evening (1800 h), is lowest in the middle of the night (0100–0200 h), and then increases until early morning—0500 h.

Indicators demonstrative of exposure to *Anopheles* bites based on human behavior [13] have been summarized in Table 5. There were 0.66 bpn occurring indoors for an unprotected individual (including periods when an individual cannot be under an ITN). The proportion of vector bites occurring while asleep for an unprotected individual is 0.27 bpn. Overall, there were 0.24 bpn prevented by using an ITN. Even ITN-protected individuals are exposed to mosquito bites when awake and this proportion of remaining exposure occurring indoors for a user of an ITN is 0.55 bpn while the proportion of exposure occurring outdoors for a protected individual is 0.45 bpn. Meanwhile, the proportion of exposure prevented by current levels of ITN use in the population is relatively low (12%), resulting in spaces and times when people are not under ITN protection or when ITNs cannot be used.

**Table 5:** Human exposure patterns to *Anopheles* bites.

Indicator	*Anopheles* bites
Directly measured biting	
• Proportion biting indoors	0.51
Behavior-adjusted exposure–unprotected individual	
• Proportion of vector bites occurring indoors for an unprotected individual	0.66
• Proportion of vector bites occurring while asleep indoors for an unprotected individual	0.27
Exposure prevented by ITN use	
• Proportion of all vector bites prevented by using an ITN	0.24
Remaining exposure for an ITN-user	
• Proportion of remaining exposure occurring indoors for a protected user of an ITN	0.55
• Proportion of remaining exposure occurring outdoors for a protected user of an ITN	0.45
Population mean exposure based on observed level of ITN use	
• Proportion of exposure prevented by current levels of ITN use in the population	0.12

**Fig 5:**
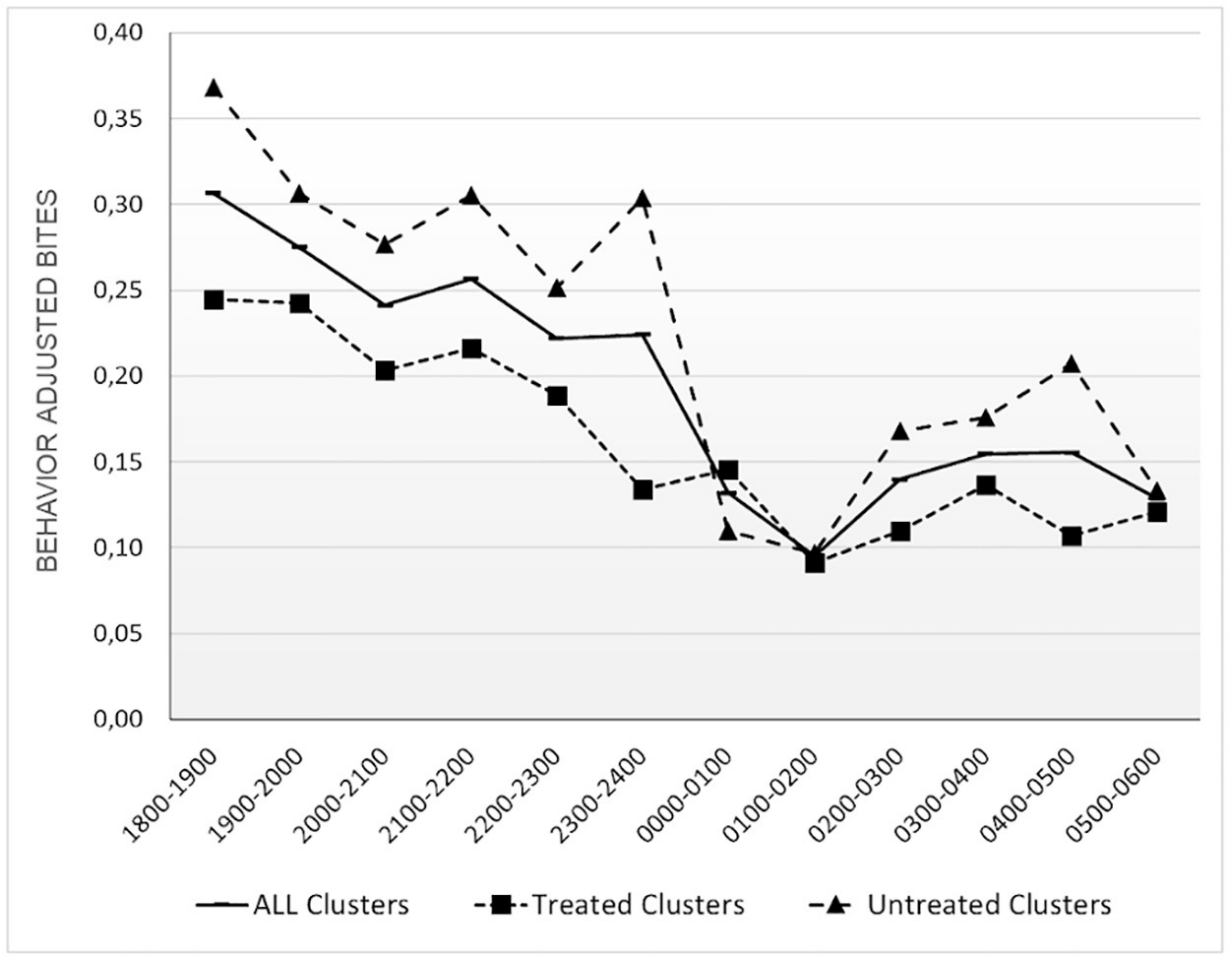
Overall behavior adjusted exposure. Overall behavior adjusted exposure over the course of a night for treated, untreated and all clusters together. Treated clusters demonstrate less exposure overall with key differences seen during times when people are indoors and not under ITN protection, that consistent with expectations from a spatial repellent product.

There was a difference in behavior adjusted bites seen in treated and untreated clusters, the sum of behavior adjusted bites per person for untreated clusters was higher than treated clusters (Fig 5).

When looking at spatial exposure indoors, outdoors when awake, and outdoors when asleep in the untreated arm, there were 1.36, 0.93 and 0.41 behavior adjusted bpn respectively. All these HBO-based rates were lower in the treated arm (1.10, 0.61 and 0.23 indoors, outdoors when awake, and outdoors when asleep respectively). Proportionally, spatial exposure was higher indoors in the treated arm (57% in the treated arm versus 50% in the untreated arm), but lower in both outdoor behavioral spaces (Outdoors when awake: 31% treated versus 35% untreated; outdoors when asleep: 12% treated versus 15% untreated). Adjusted exposure in the treated clusters was 1.94 bpn while that in the untreated clusters were 2.7 bpn–representing approximately 28% less exposure in the treated arm over the course of a night.

## Discussion

The impact of a spatial repellent on malaria incidence was evaluated in a cluster-randomized, double-blinded, placebo-controlled trial, in Sumba, Indonesia [21]. Temporal and routine indoor and outdoor HLC sampling, as part of this parent study, enabled the documentation of *Anopheles* mosquito landing rates while parallel HBO collections (corresponding to five HLC periods) recorded the spatial and temporal presence of humans along with ITN usage. Household surveys documented house construction, the presence of animals, intervention usage, and other potential drivers of malaria.

The open nature of all four types of characterized house types in Sumba–especially the presence of open eaves in 98.5% of houses, enable entry and exit of vectors. Mosquito access to humans inside houses is evident by the similar entomological landing rates both inside and outside houses. Open eaves are documented entry points for malaria vectors [32,33] and may significantly contribute to indoor biting in this area. House design has been documented to impact disease [34–39], and even in areas with high ITN coverage and usage, primary exposure to malaria may occur indoors [40,41]. The openness of the houses with consistent airflow may have also impacted the protective efficacy of the spatial repellent intervention. The similar indoor and outdoor landing rates documented in multiple vector species suggest that indoor interventions like ITNs may impact species that feed both indoors and outdoors [42,43]. Biting throughout the night, including when people are outdoors and inside before going to bed, suggests the need for supplementary protection measures in addition to ITNs.

The presence of animals inside houses, a cultural aspect of Sumba [28,44] was also associated with increased mosquito entry. Interestingly, the number of humans present was not found to be related to the number of mosquitoes indoors. Most *Anopheles* species found here feed on both humans and animals but are generally considered zoophagic [23] potentially explaining this outcome. The presence of indoor fires (cooking) or animals outside did not impact mosquito entry.

Human behavior by itself was a driver of exposure to mosquito bites. Over the course of a night, people spent time both indoors and outdoors. There was almost no mosquito personal protection in use other than ITNs. Overall, the population evaluated spent 27% of the night under ITNs, while approximately 35% of the night was spent outdoors and 38% indoors unprotected. Though this site did not have any ITN access issues (there was a mass ITN distribution in 2018) [21], ITN presence and usage was both house and cluster specific demonstrating that, unconnected to intervention presence, household behaviors will impact exposure to mosquito bites. This points to the value of passive interventions, like the spatial repellent product evaluated in the primary trial [21], where human compliance, a large factor in determining intervention efficacy, can be mitigated. Other behaviors that increased exposure included outdoor sleeping as well as indoor activities, such as cooking, where ITNs cannot be used. Appropriate social and behavior change communication (SBCC) directed at increasing ITN use both indoors and outdoors may reduce a proportion of this exposure. Relating setting-specific human behaviors with complementary entomological and epidemiological interventions that function within the spaces and times of exposure may better impact disease transmission [45].

Analyzing mosquito landing rates with human behavior data enables the spatial and temporal estimation of where and when exposure to *Anopheles* bites occurs based on intervention (ITN) presence and usage [12,15]. Human behavior and ITN use were found to impact exposure considerably relative to vector-biting-only estimations of exposure. Even though vector biting based on HLCs, peaked at the beginning of the night, midnight and then early in the morning, human behavior adjusted exposure was found to predominate early in the evening, and present throughout the night. Reduced human behavior adjusted exposure was based on people being indoors and under ITNs, and away from outdoor exposure. Over the night, most exposure (53%) occurred when people were indoors and not under the protection of ITNs (asleep or awake) followed by exposure outside (44%). This outcome immediately points to the possibility of reducing indoor exposure by increasing ITN use when asleep, while using complementary interventions like spatial repellents to combat remaining exposure occurring indoors. Alongside this observation and the use of HBO’s, the spatial repellent (SR) arm had decreased human behavior adjusted exposure in all spaces (inside and outside), though it may have proportionally increased exposure indoors, possibly attributed to people wanting to be in a space with the least biting and possibly using ITNs less. Evidence on the impact of spatial repellents on the use of other interventions (ITNs) and human behavior with ensuing remedial measures need to be evaluated.

Towards understanding the impact of human behavior on spatial repellent protection, intervention (treated) and placebo (untreated) clusters were analyzed separately with regards to adjusted exposure. Interestingly, although there was not a significant impact on vector biting rates by themselves [21], there was approximately 28% less human behavior adjusted bites in the intervention arm that had the spatial repellent over the course of the night during the period that HBOs were documented. Key differences are seen during times when people are awake indoors and not under ITN protection that consistent with expectations from a spatial repellent product. This observation is particularly important since, when looking at HLC clusters only, the primary study [21] documented a statistically inconclusive (16.4%) reduction of bites in the intervention clusters, with a statistically significant epidemiological impact (60% protective efficacy). This higher-than-expected epidemiological outcome relative to a smaller entomological impact may be explained by the impact of human behaviors on exposure as documented above i.e., actual exposure to bites was not reflected by HLC data alone. In addition, the 60% protective efficacy seen in the primary study [21] was in children under five who generally have a higher ITN use as compared to the overall population documented in this analysis. Here a greater than 28% impact on exposure in this under five cohort would be expected that would better relate to the 60% protective efficacy seen.

Characterized gaps in protection are outdoor exposure as well as exposure indoors such as when awake, and when asleep and not using ITNs. Analysis incorporating human behaviors indicate that overall, ~55% of exposure occurs indoors, suggesting that the protection from increasing ITN use can be augmented by paradigms such as spatial repellents that function when ITNs cannot be used. In this study spatial repellents fill specific gaps in protection, primarily indoors when people are not under the protection of ITNs. Overall, only about 24% of exposure is mitigated by ITN use in these communities. Even when using ITNs, 55% of remaining or residual exposure occurs indoors and 45% occurs outdoors pointing to the need for additional protective measures. Continuing gaps in exposure even with optimal ITN and spatial repellent use includes bites that continue to happen outdoors as well as indoors due to the open housing structure, limitations with the spatial repellent product, ITN durability, and so on. Solutions here may include more improved housing, evidence-based SBCC, improved intervention products, or increased complementary epidemiological interventions (e.g., mass drug administration or increased testing and treatment towards reducing the parasite reservoir).

Study limitations include the HBOs not extending through the entire timeline of the primary study which would have enabled the better evaluation of human behavioral components of protection and exposure–the limited timeline however still does demonstrate HBO-based differences in the two arms of the study. The evaluation of behaviors of children under five (the cohort followed in the primary study [21]) would have allowed for determining a relationship between protective efficacy and entomological (adjusted) exposure as well. In addition, the high heterogeneity in entomological landing rates (two separate clusters, clusters 2 and 16, were outliers in the number of mosquitoes collected) were possible confounders.

This analysis points to the importance of using HBOs both towards understanding gaps in protection as well as how interventions are being evaluated and may serve as a proxy for entomological outcomes when and if characterized in relationship to measured entomological and epidemiological endpoints. To mitigate ongoing transmission, understanding context specific spatial and temporal exposure based on the interactions of vectors, humans and interventions would be vital for a directed evidence-based control or elimination strategy.

## Supporting information

S1 File HLC questionnaire. (DOCX)

S2 File HBO questionnaire. (DOCX)

S3 File Household and bed net questionnaire. (DOCX)
