# Supplementary material for: Human behavior determinants of exposure to Anopheles vectors of malaria in Sumba, Indonesia
Source: PLoS One. 2022 Nov 14;17(11):e0276783. doi: 10.1371/journal.pone.0276783 (PMC9662732; doi:10.1371/journal.pone.0276783)
Supplement: S1 File — (DOCX) [file pone.0276783.s001.docx]

# HLC Questionnaire

Questions:

- - - 1. Any fire burning/smoke outside the house from 18:00 – 24:00 during HLC? (Yes/No)
      2. Any fire burning/smoke outside the house from 24:00 – 6:00 during HLC? (Yes/No)
      3. Any fire burning/smoke inside the house from 18:00 – 24:00 during HLC? (Yes/No)
      4. Any fire burning/smoke inside the house from 24:00 – 6:00 during HLC? (Yes/No)
      5. Any large animals outside the house from 18:00 – 24:00 during HLC? (Yes/No)
      6. Any large animals outside the house from 24:00 – 6:00 during HLC? (yes/No)
      7. Any large animals inside/under the house from 18:00 – 24:00 during HLC? (Yes/No)
      8. Any large animals inside/under the house from 24:00 – 6:00 during HLC? (Yes/No)
      9. How many people slept in the house during HLC?
      10. How many people slept under bednet in house during HLC?
      11. Any efforts to avoid mosquito bites used during HLC (multiple choice)
      12. Notes if any (optional)
