# Supplementary material for: Human behavior determinants of exposure to Anopheles vectors of malaria in Sumba, Indonesia
Source: PLoS One. 2022 Nov 14;17(11):e0276783. doi: 10.1371/journal.pone.0276783 (PMC9662732; doi:10.1371/journal.pone.0276783)
Supplement: S2 File — (DOCX) [file pone.0276783.s002.docx]

# HBO questionnaire

Questions: (asked hourly during HLC)

- - - 1. How many people under net indoor?
      2. How many people NOT under net indoor?
      3. How many people awake outdoor?
      4. How many people asleep outdoor?
