# Supplementary material for: Human behavior determinants of exposure to Anopheles vectors of malaria in Sumba, Indonesia
Source: PLoS One. 2022 Nov 14;17(11):e0276783. doi: 10.1371/journal.pone.0276783 (PMC9662732; doi:10.1371/journal.pone.0276783)
Supplement: S3 File — (DOCX) [file pone.0276783.s003.docx]

# Household and bed net questionnaire

1. Building structure information
2. Wall material (multiple choice)
3. Roof material (multiple choice)
4. Floor material (multiple choice)
5. Distance floor to the land (cm)
6. Eaves opened (Yes/No)
7. Window number (screened or not)
8. Door number
9. Fire burning inside the house (Yes/No)
10. Fire burning outside the house (Yes/No)
11. Electrical source (Yes/No)
12. Protection against mosquito bites
13. Last time indoor residual spraying (IRS) (multiple choice)
14. Used of mosquito protection (multiple choice)
15. Bednet number
16. Livestock
17. Type and number of livestock inside the building
18. Type and number of livestock outside the building
19. Bednet condition
20. The origin of the bednet is obtained (multiple choice)
21. Age of the bednet
22. Insecticide net (Yes/No)
23. Brand of the bednet (multiple choice)
24. Bednet condition (multiple choice)
25. The bednet hung last night (Yes/No)
